# Supplementary material for: Complement Component C1q as a Potential Diagnostic Tool for Myalgic Encephalomyelitis/Chronic Fatigue Syndrome Subtyping
Source: J Clin Med. 2021 Sep 15;10(18):4171. doi: 10.3390/jcm10184171 (PMC8467657; doi:10.3390/jcm10184171)
Supplement: Supplementary file 1 [file jcm-10-04171-s001.zip › jcm-1377046-supplementary/Supplementary Figure S1_with legends.pdf]

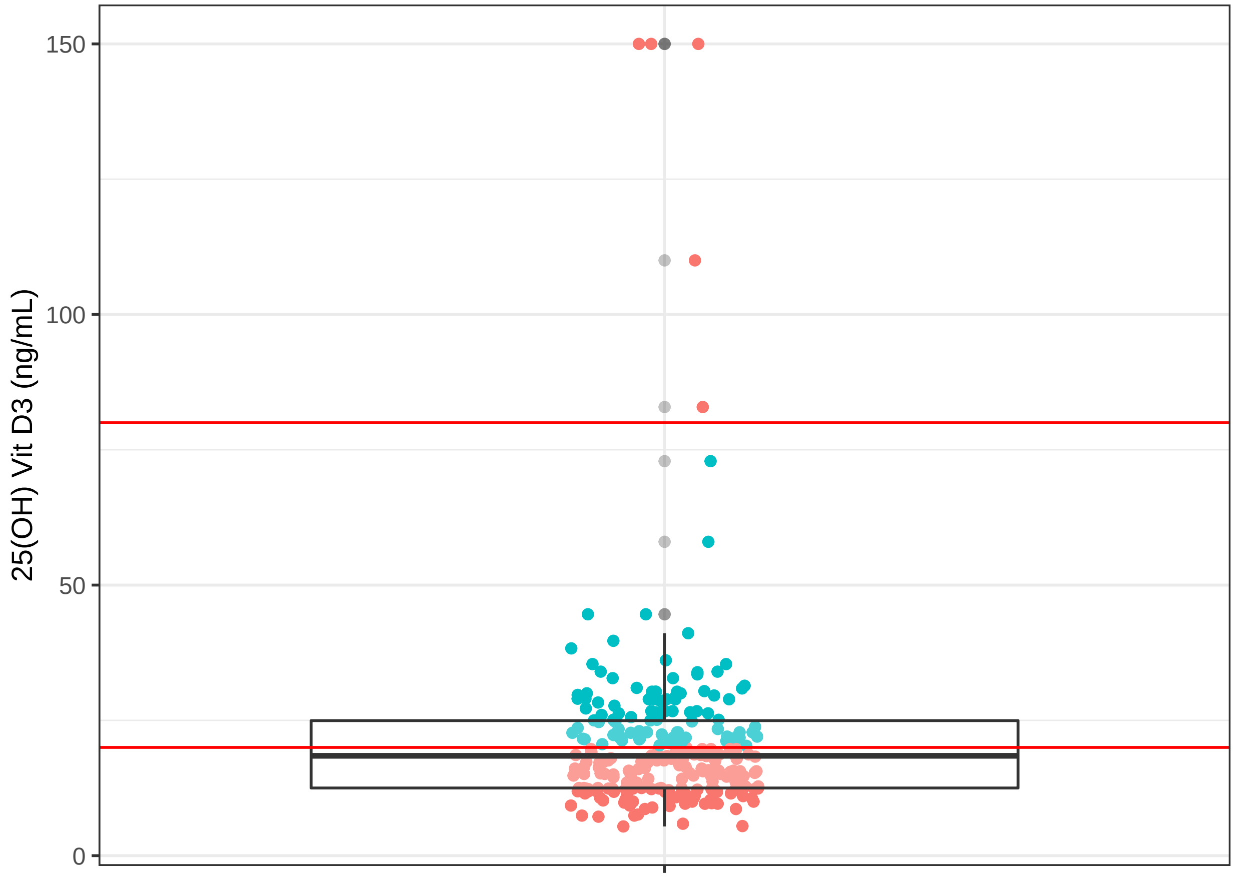

Supplementary Figure S1. Box plots of top blood analytic variables showing off-normal reference values in our cohort (n = 250). Red lines indicate normal range values. Black line within the box is the median within quartile values. Normal values are shown in green while abnormal values are shown in red.

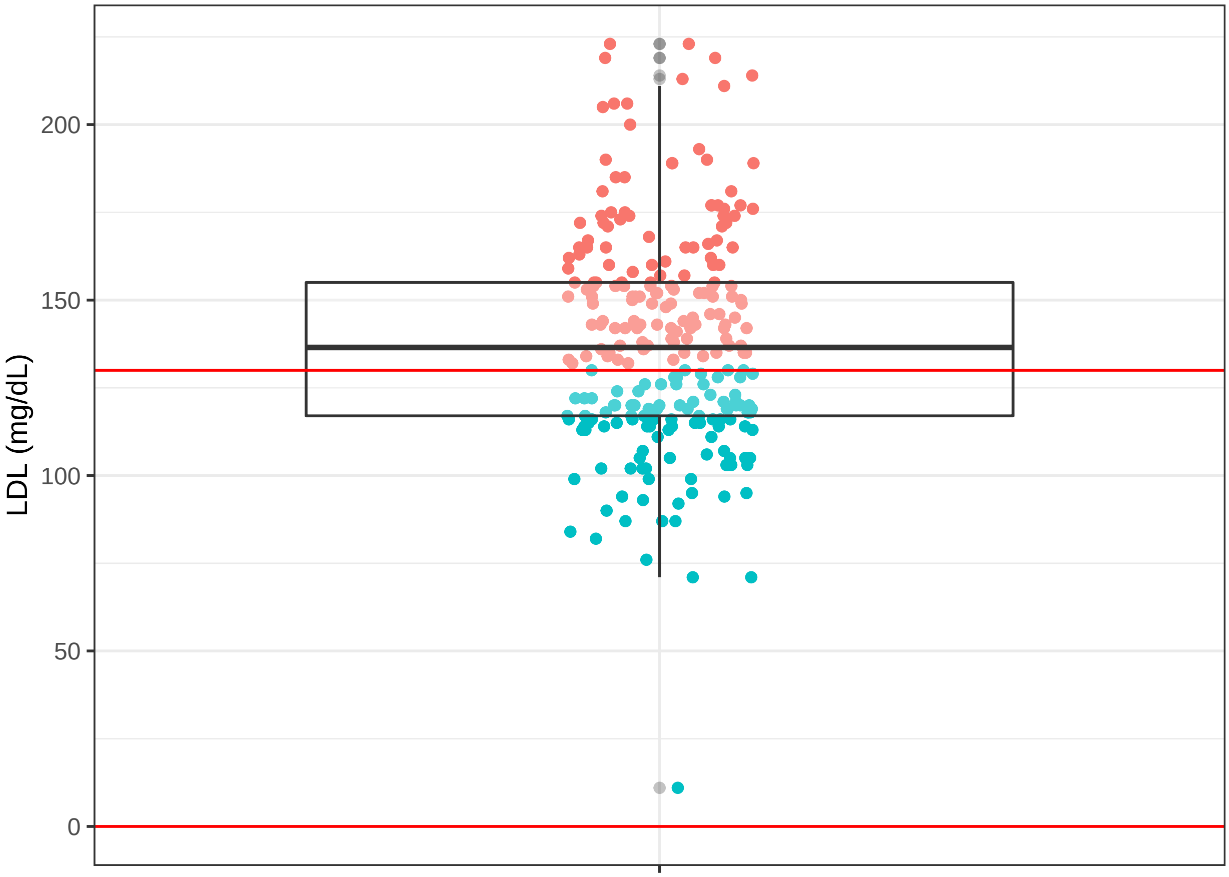

Supplementary Figure S1. Box plots of top blood analytic variables showing off-normal reference values in our cohort (n = 250). Red lines indicate normal range values. Black line within the box is the median within quartile values. Normal values are shown in green while abnormal values are shown in red.

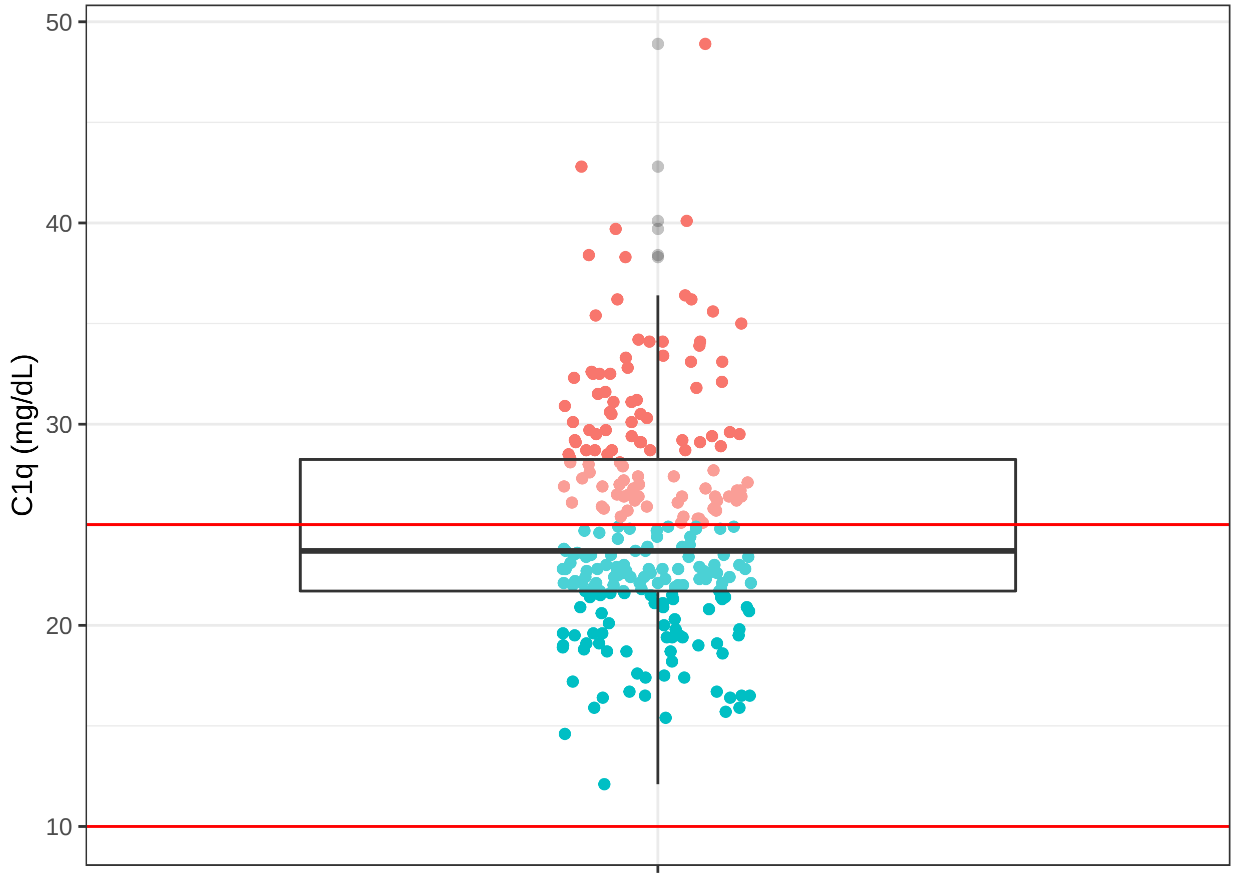

Supplementary Figure S1. Box plots of top blood analytic variables showing off-normal reference values in our cohort (n = 250). Red lines indicate normal range values. Black line within the box is the median within quartile values. Normal values are shown in green while abnormal values are shown in red.

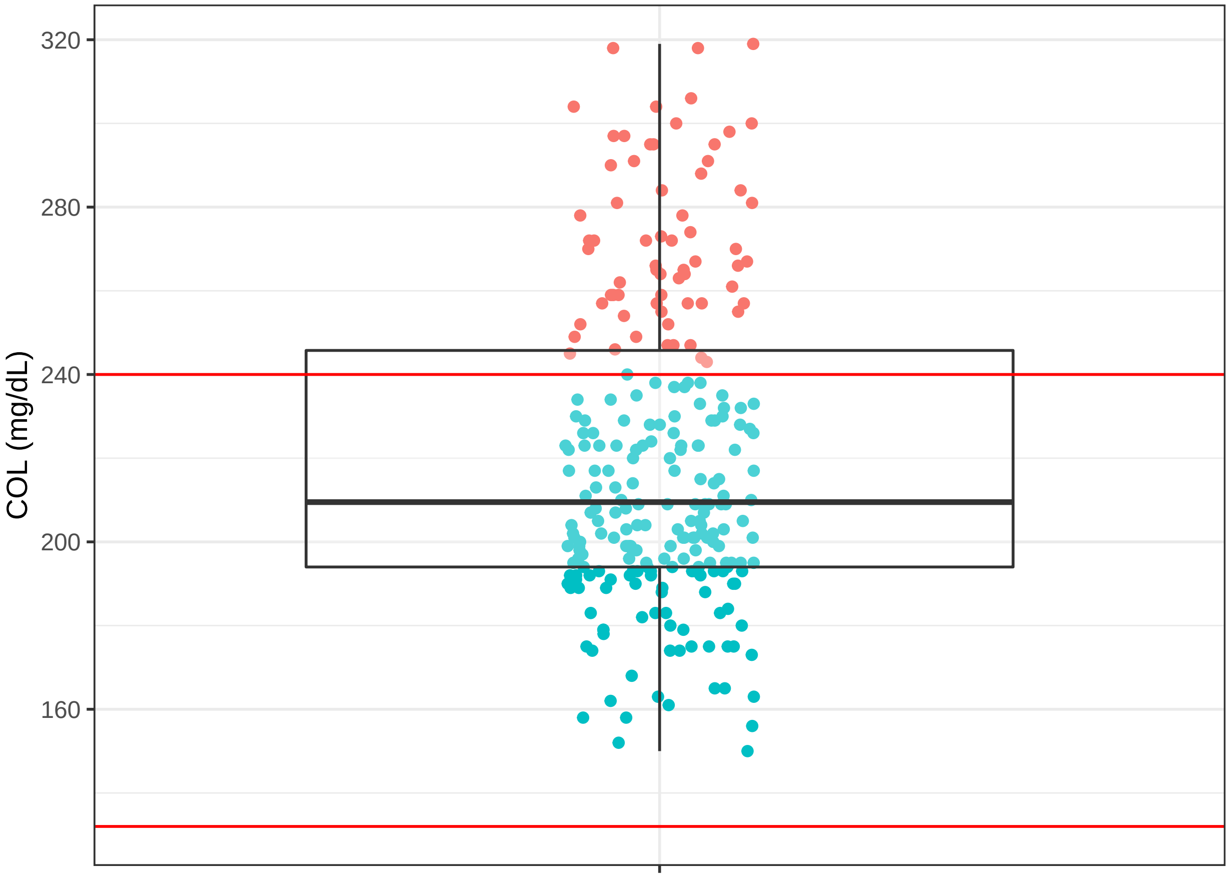

Supplementary Figure S1. Box plots of top blood analytic variables showing off-normal reference values in our cohort (n = 250). Red lines indicate normal range values. Black line within the box is the median within quartile values. Normal values are shown in green while abnormal values are shown in red.

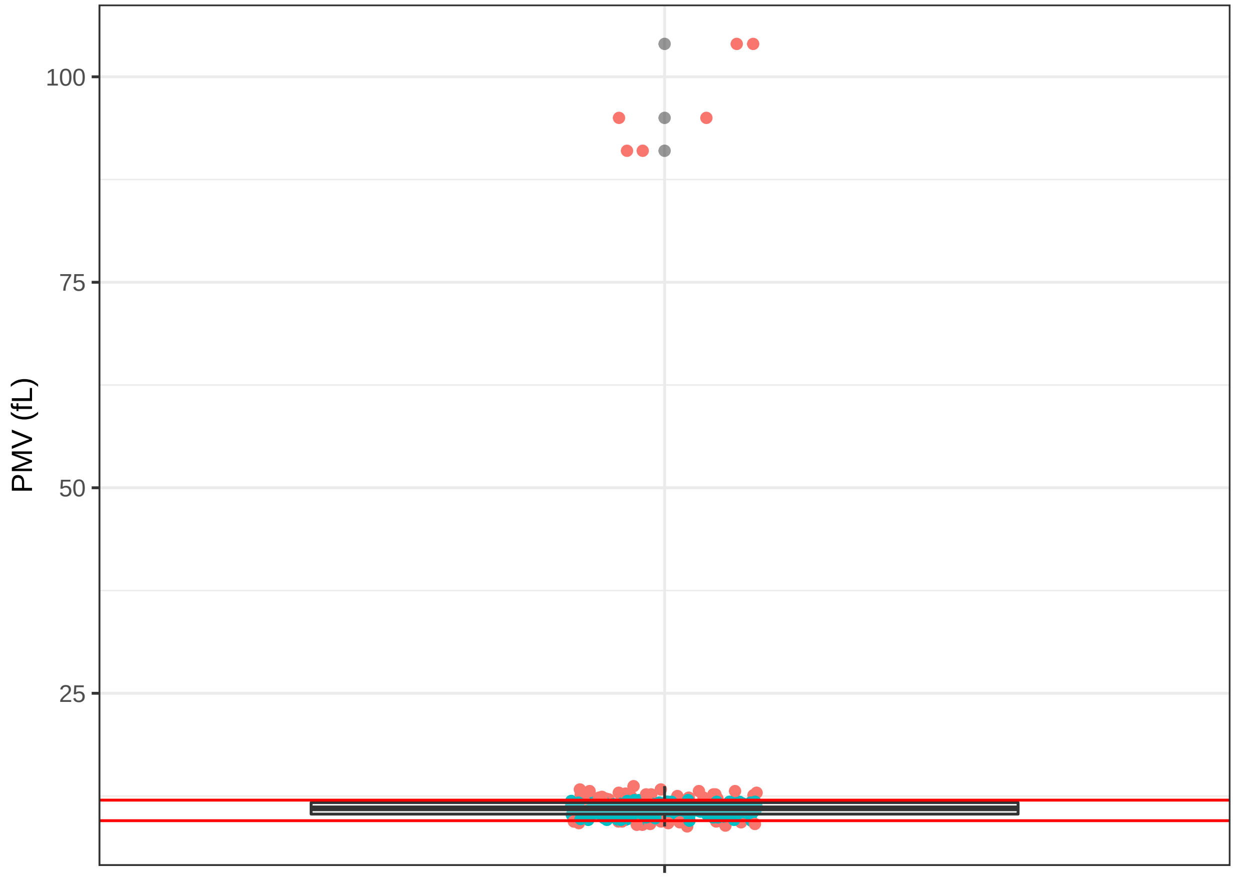

Supplementary Figure S1. Box plots of top blood analytic variables showing off-normal reference values in our cohort (n = 250). Red lines indicate normal range values. Black line within the box is the median within quartile values. Normal values are shown in green while abnormal values are shown in red.
